# Comparison of groups with different forms of problematic Internet use pro-actively recruited in the setting of vocational schools

**DOI:** 10.1186/1940-0640-10-S2-O7

**Published:** 2015-09-24

**Authors:** Anja Bischof, Gallus Bischof, Luisa Hauer, Lea Braden, Hans-Juergen Rumpf

**Affiliations:** 1 Center for Integrative Psychiatry, University of Luebeck, Luebeck, Germany

## Background

Previous studies found adolescents and young adults to have a higher vulnerability to develop problematic or pathological Internet use. The project PINTA-Diari revealed that in spite of the limitation on “Internet gaming” in the DSM-5, users of social networks showed criteria for problematic Internet use, too. The present study examines problematic Internet use and other harmful behavior in the setting of vocational schools.

## Material and methods

In two vocational schools in Luebeck, Germany, 1209 students were systematically screened with the Compulsive Internet Use Scale (CIUS), the Alcohol Use Disorders Identification Test-Consumption (AUDIT-C), the Mental Health Inventory (MHI-5) and other validated questionnaires. Students were included in the analyses when scoring at least 21 on the CIUS-scale for problematic Internet use (n=313) and were split up in three groups: Online gamers (n=50), social networker (n=135) und others (n=124).

## Results

Of the sample, 32.3% with problematic Internet use were screening-positive on the AUDIT-C, 34.7% were daily smokers, 20% consumed marihuana und 8.7% other illegal drugs. The guidelines for fruit and vegetable consumption (“Five a day”) were met by only 11.2% of the students. No group differences between online gamers, social networkers, and users of other applications could be found except in mental health where online gamers performed better than users of other applications (MHI-5: gamers M 13,8 [SD 3,0]; others M 11,8 [SD 3,6], adjusted for sex: p=.021) but did not differ significantly from users of social networks.

## Conclusions

Our data support recent findings that users of other Internet applications do not differ significantly from online gamers in a number of characteristics and that there is a need for further research and brief interventions. Students of vocational schools are a high-risk group concerning problematic Internet use and substance use. The problem groups can be easily identified in the setting by proactive screening measures.

